# Bioconversion of inorganic selenium to less toxic selenium forms by microbes: A review

**DOI:** 10.3389/fbioe.2023.1167123

**Published:** 2023-03-13

**Authors:** Xinling Nie, Xurui Yang, Junyi He, Pei Liu, Hao Shi, Tao Wang, Daihui Zhang

**Affiliations:** ^1^ Faculty of Life Science and Food Engineering, Huaiyin Institute of Technology, Huaian, China; ^2^ Jiangsu Provincial Engineering Laboratory for Biomass Conversion and Process Integration, Huaiyin Institute of Technology, Huaian, China; ^3^ Department of Microbiology, The University of Georgia, Athens, GA, United States; ^4^ Institute of Chemical Industry of Forest Product, Chinese Academy of Forestry, Nanjing, Jiangsu, China

**Keywords:** feed additives, selenium, selenium enrichment, selenium nanoparticles, synthetic mechanism

## Abstract

In recent years, microbial conversion of inorganic selenium into an efficient and low-toxic form of selenium has attracted much attention. With the improvement of scientific awareness and the continuous progress of nanotechnology, selenium nanoparticles can not only play the unique functions of organic selenium and inorganic selenium but also have higher safety, absorption and biological activity than other selenium forms. Therefore, the focus of attention has gradually shifted beyond the level of selenium enrichment in yeast to the combination of biosynthetic selenium nanoparticles (BioSeNPs). This paper primarily reviews inorganic selenium and its conversion to less toxic organic selenium and BioSeNPs by microbes. The synthesis method and potential mechanism of organic selenium and BioSeNPs are also introduced, which provide a basis for the production of specific forms of selenium. The methods to characterize selenium in different forms are discussed to understand the morphology, size and other characteristics of selenium. In general, to obtain safer and higher selenium content products, it is necessary to develop yeast resources with higher selenium conversion and accumulation.

## 1 Introduction

Selenium is an essential trace element for organisms, and it mainly exists in the inorganic form in nature (Kieliszek and Błażejak, 2013; Kieliszek et al., 2016). Although selenium is a necessary element in living organisms, the appropriate concentration of inorganic selenium supplementation is close to its toxic concentration. Slightly exceeding unnecessary nutrient levels can lead to poisoning, so its use must be strictly controlled (Goldhaber, 2003; Long et al., 2020; Lv et al., 2021). The use of inorganic selenium was restricted because of its toxicity until the bioactive selenium was found to be in the active center of glutathione peroxidase (GSH-PX) in rats.

Selenium participates in a variety of physiological metabolic processes in living organisms. Generally, the intake of selenium in humans and animals is very low. Inorganic selenium is rapidly metabolized and difficult to be absorbed, so its bioavailability is directly related to the chemical form of selenium. Organic selenium has been reported to persist longer in humans and animals and to be less toxic to the host (JäGer et al., 2016; Kim and Kil, 2020). At the same time, organic selenium has higher bioavailability and safety, and its active efficiency is approximately seven times higher than that of inorganic selenium (Hadrup and Ravn-Haren, 2021; Jiang et al., 2021).

Organic selenium is a dietary supplement resource for the feed and dietary supplement industries. Selenomethionine (SeMet) is the main source in plants, and selenocysteine is the main source in animals and humans. In addition, they exist in organisms in the form of selenoproteins, selenopolysaccharides, selenoamino acids, selenonucleic acids, selenolipids and their derivatives. At present, biological selenium supplementation is mainly occurs through plants growing in selenium-containing soil (Chupani et al., 2017), selenium-rich feed to produce selenium-rich functional livestock and poultry products (Saffari et al., 2017; Wang J. et al., 2018), and microbes (especially yeast) cultured in selenium-rich medium, which have greatly improved the efficiency of selenium uptake (Fairweather-Tait et al., 2011; Răduță and Curcă, 2017). In addition, organic selenium has a positive effect on the health and growth of fish at small concentrations, and thus selenium-enriched products such as seleno-amino acids, selenium-enriched yeasts and selenium polysaccharides have recently been gradually applied to aquaculture (Guimaraes et al., 2021). Except for organic selenium, selenium nanoparticles (SeNPs, selenite or selenate is reduced to Se^0^), especially biosynthesized SeNPs (BioSeNPs), have been recently confirmed to be less toxic and more beneficial to the host (Bhattacharjee et al., 2019; Kumar and Prasad, 2021). Therefore, they are considered a better alternative for selenium supplementation.

## 2 Organic selenium from various organisms

### 2.1 Organic selenium from plants

Selenium is not an essential element for plant growth, but plants are the main source of selenium supplementation for humans and animals. As the selenium content in the soil decreases, the amount of selenium available to organisms from selenium-enriched plants gradually decreases (Turło et al., 2010). To solve the selenium deficiency problem and improve the selenium content in the growing environment of plants, some measures are usually adopted, such as increasing the selenium content in the soil, spraying the plants with selenium solution, and using the root tissue of plants to realize the conversion of selenium.

Selenium is mainly taken up by plants in the forms of Se (Ⅳ), Se (Ⅵ), HSeO^3-^, HSeO_3_, and organic selenium, such as selenocysteine (SeCys) and SeMet, *via* the sulfur assimilation pathway. There may be multiple pathways involved in selenium metabolism in selenium-supplemented plants: including major sulfur/nitrogen metabolism, hormonal regulation, redox metabolism, and transcriptome changes in secondary metabolite biosynthesis, which affect not only the accumulation of metabolites but also the type and level of selenium accumulation, affecting its utilization in the human body. It is known that the accumulation, toxicity and detoxification effects in different plant tissues vary significantly depending on their morphology, and there is also a large difference in the ability to accumulate selenium in their root and stem tissues (Lidon et al., 2018; Shahid et al., 2018). Plants were divided into selenium-accumulating plants (selenium main indicator plants) and selenium non-accumulating plants (selenium secondary indicator plants). Primary selenium-accumulating plants mostly grow in selenium-rich areas, while selenium-non-accumulating plants can grow in selenium-rich or selenium-deficient soils (Thiry et al., 2012; Winkel et al., 2015; Michalke, 2018). Among several plant food sources of selenium supplementation, Brazil nuts grown in selenium-containing soil in the Brazilian Amazon region show the highest selenium content at 19.2 μg/g. Astragalus, sunflower, cauliflower, some varieties of cabbage, onion, garlic, and cereals (rice, wheat) are good sources for selenium accumulation, as their respective derivatives (SeMet, SeCys and SeO_3_^2-^) are rich (Prange et al., 2018; Zhou et al., 2018; Fairweather-Tait et al., 2019). For patients with selenium deficiency, the selenium contents of crops can also be increased through bioaugmentation methods (application of selenium-rich fertilizer), which is a safe and effective method for residents in selenium-deficient areas to supplement selenium (Thiry et al., 2012). Through the daily balanced supplementation of plants with high selenium content, its requirement can be basically satisfied (Hart et al., 2011).

### 2.2 Organic selenium from animals

Selenium, which is closely related to the redox reaction in animals, can prevent and treat some diseases and enhance animal immunity. Since animals cannot synthesize selenium by themselves, it is necessary to supplement appropriate doses of selenium to improve the quality of livestock and poultry products, promote the enrichment of selenium in animal tissues, and produce livestock and poultry products with selenium enrichment function (Xin and Cqg, 2022). Aquatic animal food in the human diet is a more economic and effective source of selenium (Fairweather-Tait et al., 2019). Aquatic animals with high levels of selenium include fish (tuna and salmon), oysters, and shellfish. As reported, 50% of dietary selenium supplementation in British adults comes from animal tissues (Michalke, 2018). At present, the most common method for animal selenium enrichment is to add selenium-enriched yeast to the feed. Livestock and their derivatives (meat, organs, milk, *etc.*) contain SeMet, SeCys, and SeO_4_^2-^ (D'Amato et al., 2020). The eggs and meat fed selenium-enriched grains are also rich in selenium protein. The eggs of such poultry mainly contain SeMet and SeCys (Rayman, 2008).

### 2.3 Organic selenium from microbes

It is known that the conversion of inorganic selenium by microbial method is better than others because its conversion process is rapid, efficient and environment-friendly. To solve the problem of bioremediation of selenium contamination in the environment, the microbial transformation of different forms of selenium in the environment was discussed to produce selenoproteins and selenium nanoparticles through reduction, methylation and demethylation (Eswayah et al., 2016). Selenium nanoparticles were produced by removing oxygen anions from wastewater by anaerobic microbes, and proteins or peptides were adsorbed (Gonzalez-Gil et al., 2016). Adding selenium salts (Na_2_SeO_3_, Na_2_SeO_4_, *etc.*) to microbial culture media to obtain selenium-enriched products is a common method to prepare organic selenium or selenium nanoparticles (Khatiwada and Subedi, 2021). Microbes are also good candidates for food additives, and they are able to convert inorganic selenium into organic selenium with high nutritional value (Kieliszek et al., 2015; Rayman et al., 2018). Selenium-enriched *Lactobacillus* can not only have high organic selenium conversion but also destroy the cell structure of pathogens, inhibit the growth of pathogens and show higher inhibitory activity. Approximately 50% of the inorganic selenium can be converted to organic selenium by using selenium enrichment with *Bifidobacterium*, which is sufficient to meet the homeostasis of selenium in rats; that is, the absorption, distribution, metabolism and excretion of selenium in the two forms are equal. It is mainly involved in the metabolic process and redox reaction of selenoids to maintain the balance of the environment and physiological system in rats (Loeschner et al., 2014). It has been shown that the organic selenium content of *Saccharomyces cerevisiae* ranges from 1 to 4.5 mg/g dry weight, and 58% of selenium compounds are present in the form of methyl selenocysteine (SeMCys). The final concentration of SeMCys reached 5.746 mg/g (Ye et al., 2020; Wu et al., 2021). In *Candida utilis*, another selenium-enriched yeast, the organic selenium content was 1.0 mg/g dry cell weight (Yang et al., 2013; Zhang et al., 2019). Mushrooms are rich in selenium and tend to store excess inorganic selenium in the body (Egressy-Molnár et al., 2016). Yeast assimilates inorganic selenite through sulfur metabolism (Rao et al., 2010), creating a selenide cell containing a variety of organic selenium compounds. Compared with plants, the protein content in yeast is higher (León et al., 2010). Yeast can accumulate different forms of selenium through both intracellular and extracellular bioaccumulation (Kieliszek et al., 2015). Inorganic selenium is transformed into organic forms by biotransformation (SeCys, SeMet, *etc.*), which can not only reduce toxicity but also improve the absorption dosage (Rayman, 2004; Yin et al., 2010; Thiry et al., 2013).

## 3 The green synthesis of BioSeNPs

BioSeNPs possess good stability to bind proteins or polysaccharides (Wang Y. et al., 2018; Cai et al., 2018), which has received more attention from researchers. Chemical synthesis of nano-selenium requires specific chemicals. Ascorbic acid, cysteine, sodium sulfate, *etc.*, Were used as reducing agents to prevent the aggregation of nanoparticles in the presence of stabilizers, but some residues of these chemicals limited the application of the formed selenium nanoparticles in the field of medicine (Pyrzynska and Sentkowska, 2021). Physical methods require extreme reaction conditions. The method of synthesis of selenium nanoparticles from plant extracts does not require the use of toxic chemicals and can control the size, shape and stability of the nanoparticles. SeNPs synthesized in this manner show unique potential in biomedical applications such as tumor therapy, targeted chemotherapy, molecular diagnostics, and drug delivery (Pyrzynska and Sentkowska, 2021). BioSeNPs synthesized by bacteria, fungi and plants are more environmentally friendly than those synthesized by chemical or physical approaches (Shoeibi and Mashreghi, 2017). They interact with proteins and other biomolecules containing functional groups such as NH, C = O, COO, and C-N present in plant extracts of microbial nuclei and are therefore biologically active (Husen and Siddiqi, 2014). Experiments with different forms of selenium have proven that BioSeNPs show the advantages of lower toxicity and higher absorption activity compared with other forms of selenium, and they can improve the antioxidant capacity (Bhattacharjee et al., 2019; Kumar and Prasad, 2021). BioSeNPs are widely recognized as excellent adsorbents that can not only adsorb heavy metal ions in soil and water but also recycle metal ions (Jain et al., 2016). In addition, some experiments have shown that BioSeNPs have lower toxicity, higher antioxidant capacity, better antibacterial properties and better stability than other forms of selenium and will become a new generation of selenium supplement food additives and therapeutic agents (Gangadoo et al., 2020; Nie et al., 2022). Therefore, selenium nanoparticles produced by green synthesis can be used as an alternative to antibiotics.

Many researchers have carried out relevant studies, and the results show that *Saccharomyces boulardii*, *Bacillus subtilis*, *Escherichia coli*, green algae and other organisms can reduce Se cations to Se^0^ under aerobic or anaerobic conditions without additional chemical reagents (Fesharaki et al., 2010; Song et al., 2017b; Asghari-Paskiabi et al., 2020; Li et al., 2021). In addition, BioSeNPs are able to combine with SeCys, SeMet, selenoprotein and other compounds and participate in many biological processes of organisms (Loeschner et al., 2014; Hadrup et al., 2016; Hadrup et al., 2019). They are widely used in medical treatment, biosensors and environmental remediation (Sonkusre, 2014; Song et al., 2017a; Cheng et al., 2017; Sonkusre and Cameotra, 2017). Therefore, a green, efficient and low-cost biotechnology route to convert toxic selenite into non-toxic BioSeNPs is of great significance (Wadhwani et al., 2016).

**FIGURE 1:**
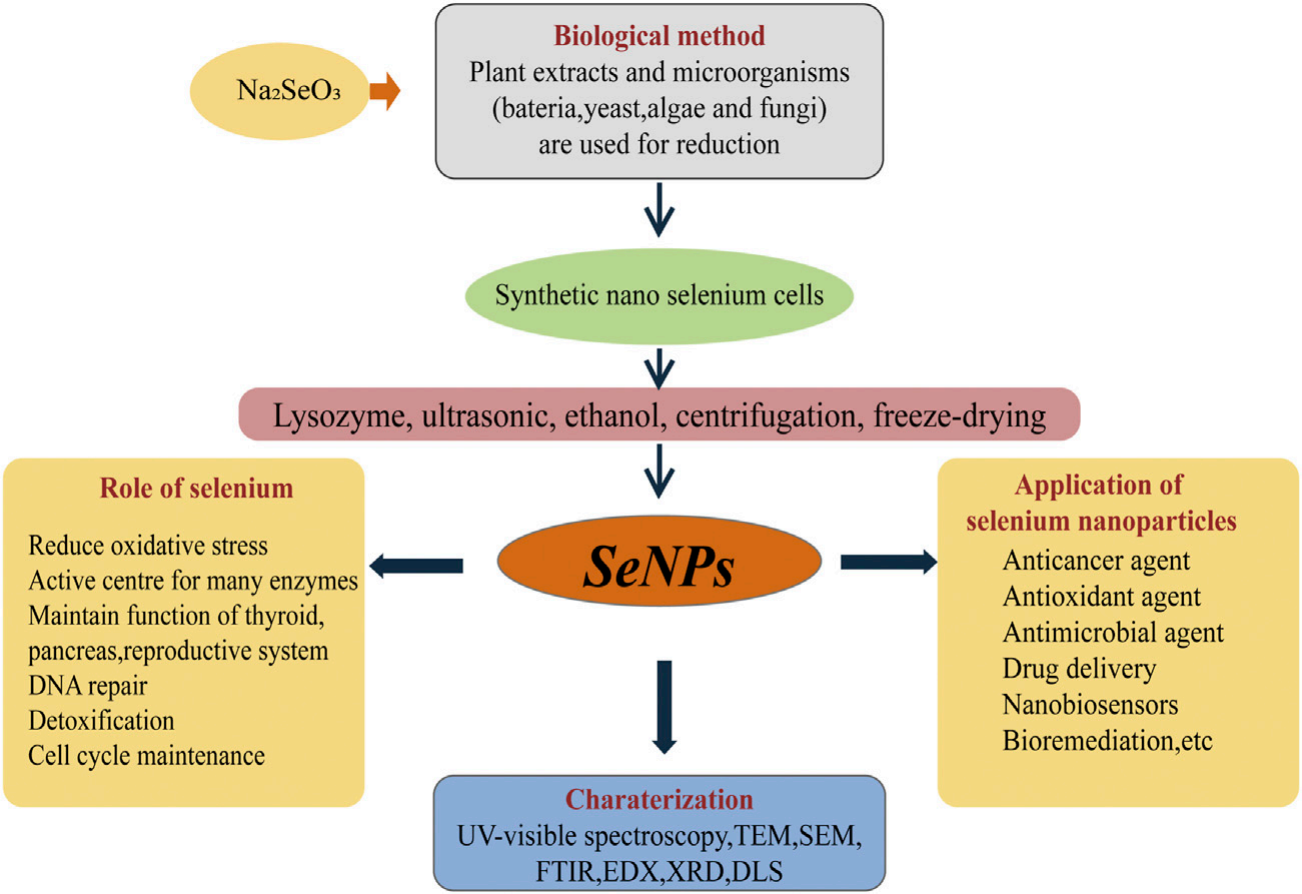
The “green synthesis” pathway of BioSeNPs and applications. Na_2_SeO_3_ was added to the fermentation medium of microbes (bacteria, yeast and fungi) to produce nano-selenium cells. The nano-selenium particles were collected by centrifugation, wall breaking, extraction and freeze-drying. Through TEM, FTIR, EDX, XRD and UV-visible spectroscopy, the morphology and content of nano-selenium can be observed and characterized. The applications of nano-selenium are broad, mainly including anticancer agents, antimicrobial agents, bioremediation sensors and so on.

Compared with chemical methods, the process of microbial synthesis of BioSeNPs belongs to “green chemistry” (Figure 1). Synthesizing BioSeNPs through biotechnology is a harmless chemical substitution method to obtain selenium. Selenite and selenate are involved in the synthesis of proteins by microbes (Hussain et al., 2016; Park et al., 2016; Prasad et al., 2016; Singh et al., 2016; Rosenfeld et al., 2017). It is well known that most microbes can reduce selenate or selenite to Se^0^.

## 4 Metabolic pathways of organic selenium and BioSeNPs

**FIGURE 2:**
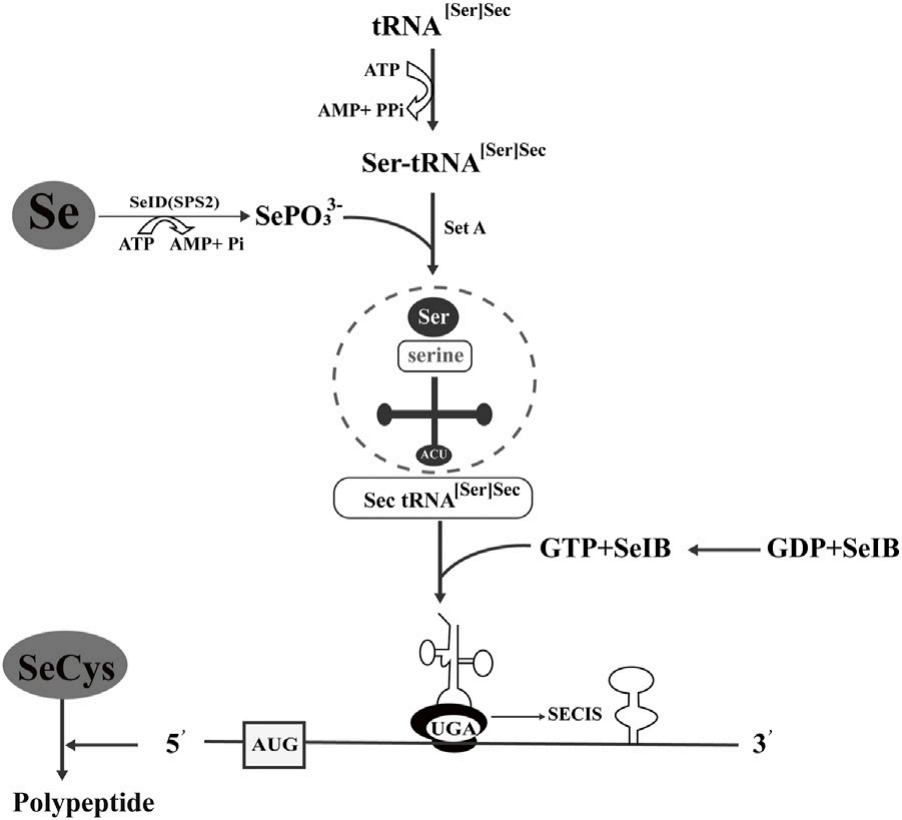
Synthesis and incorporation of selenocysteine in prokaryotes. tRNA [Ser]Sec carries SeCys and recognizes the codon of UGA. Ser-tRNA [Ser]Sec is involved in SeCys biosynthesis, and selenium phosphate synthase D/selenophosphate synthase 2 (SeID/SPS2) catalyzes selenium to form the selenium donor of monoselenophosphate (MSP). SeIB is a specific translation factor, and its C-terminal amino acid sequence of approximately 270 bp can bind with GU on the top ring of the SeCys insertion sequence (SECIS) step-loop structure.

In view of the beneficial metabolism of yeast, organic selenium metabolites can be obtained economically and efficiently by culturing yeast-produced selenoyeasts in high concentrations of inorganic selenium compounds. The synthesis and incorporation of SeCys in prokaryotes was introduced (Figure 2), and the synthetic mechanism is also suitable for yeast. When sodium selenate enters the organism, the metabolic pathway is similar to thioamino acids, and the microbe can detoxify excess selenate (Fairweather-Tait et al., 2011). The activated selenate was reduced to selenite to synthesize SeMet and SeCys. SeCys is the 21st genetically encoded amino acid translated into protein by the UGA codon (Lee et al., 1989). SeCys is integrated into the protein through a tRNA molecule with an anticodon complementary to UGA. SeCys tRNA is unique in that it controls the expression of the whole selenoprotein family (Leinfelder et al., 1988). Unlike the other 20 amino acids in the genetic code, SeCys is generally synthesized on tRNA through serine as an intermediate. For SeCys biosynthesis, a version of selenium phosphate synthase 2 (SPS2) containing cysteine is also required to directly participate in the generation of selenium donor phosphate monosilene phosphorylated tRNA, which in turn acts as the substrate of SPS2 and finally generates SeCys. γ-Glutamic acid, selenium, cysteine, and glycine form glutathione under the catalysis of glutathione synthase. In this pathway, SeMet and SeCys reflect the degree of substitution of selenium for sulfur. Selenium mainly exists in the form of SeCys (González-Salitre et al., 2021).

**FIGURE 3:**
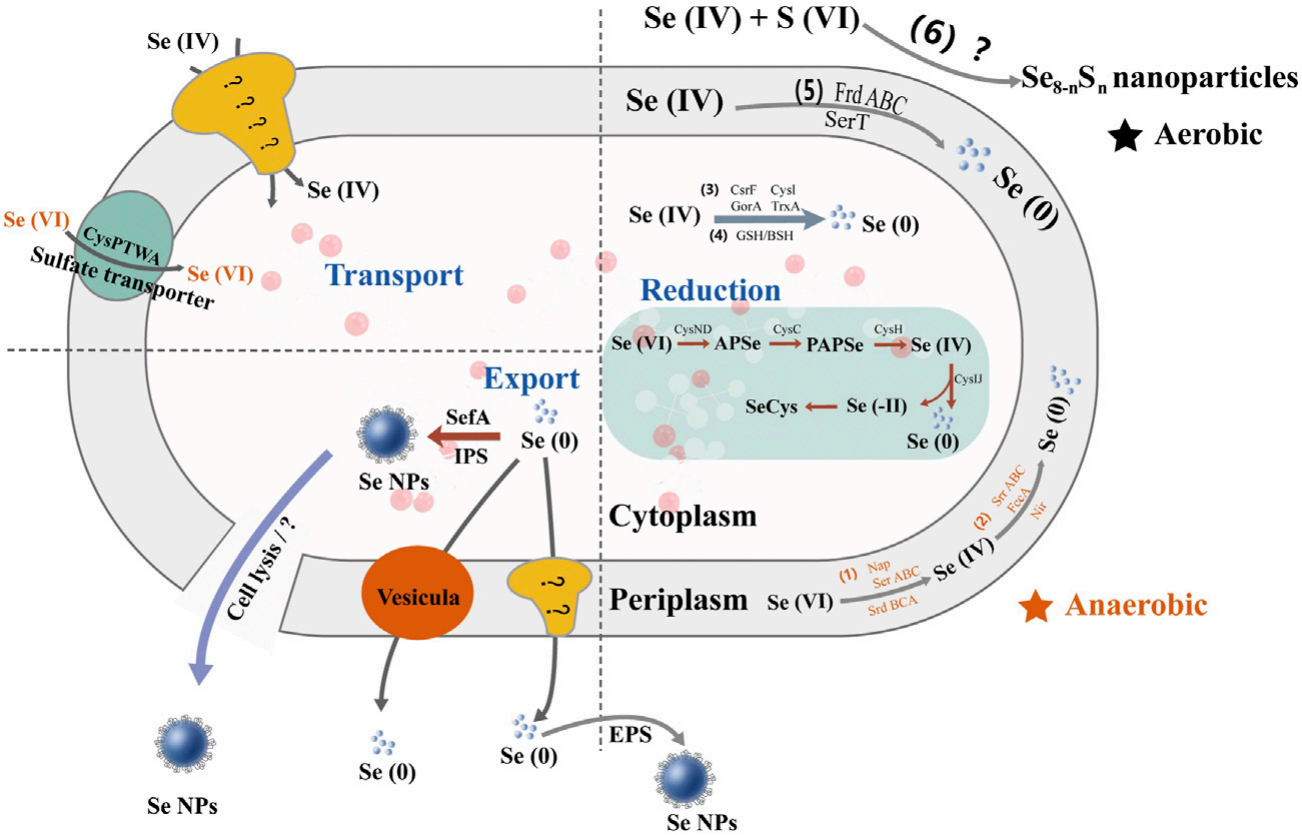
The multiple metabolic pathways of selenate and selenite in bacteria. Se(VI) is anaerobically reduced to Se(IV) in the periplasm by respiratory selenate reductase (Srr) and nitrate reductase (Nir) and further converted to BioSeNPs by different reductases, e.g., SrrABC, fumarate reductase (FccA), Nir, and selenite reductase (SerT). It also hydrolyzes glutathione (GSH) and benzenesulfonyl (BSH) to BioSeNPs.

**FIGURE 4:**
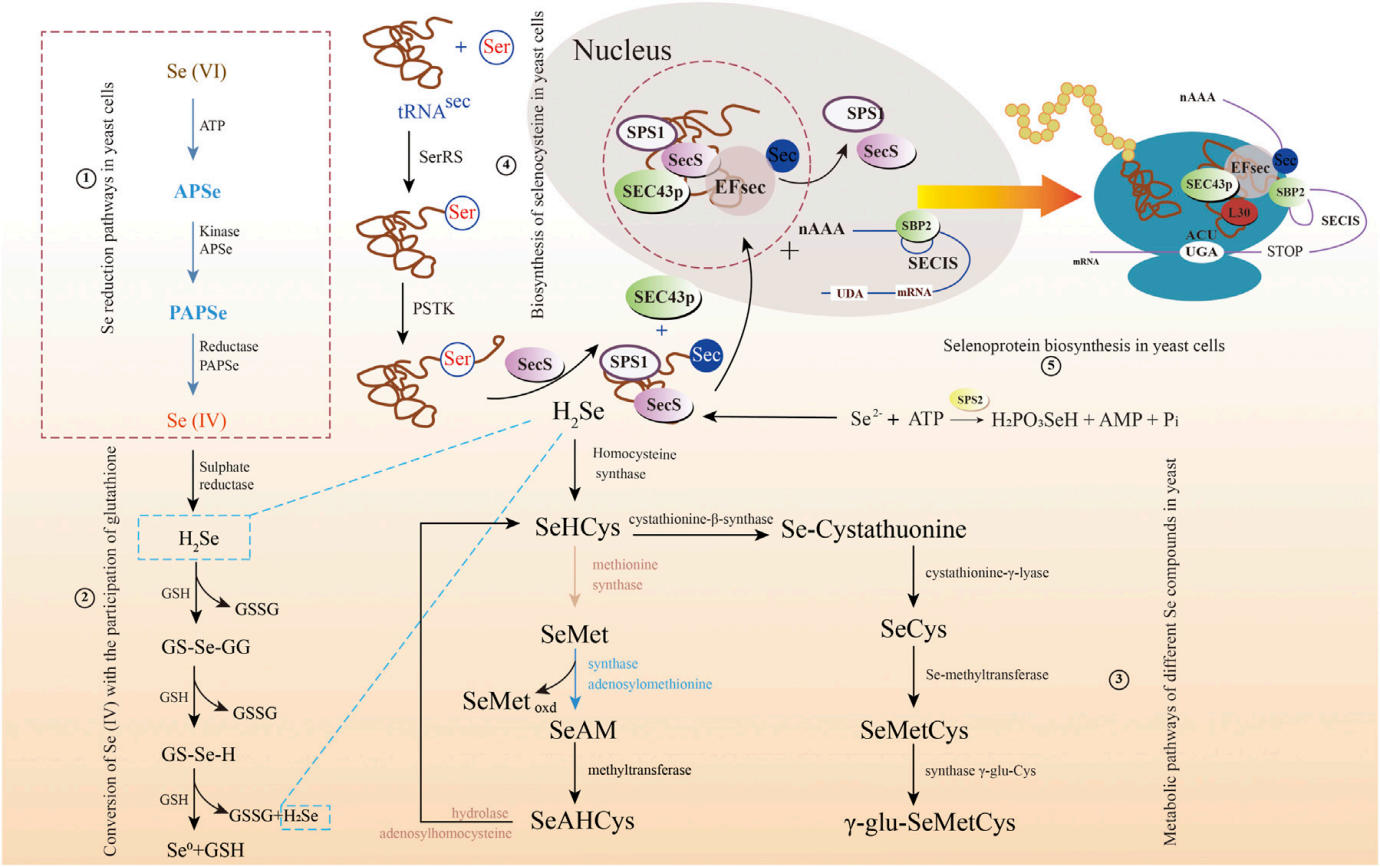
The multiple metabolic pathways of selenate and selenite in yeast. 1) Selenium reduction pathways in yeast cells; 2) Conversion of Se(IV) with the participation of glutathione; 3) Metabolic pathways of different selenium compounds in yeast; 4) Biosynthesis of selenocysteine in yeast cells; and 5) Selenoprotein biosynthesis in yeast cells.

Understanding the metabolic pathway of selenium is of great significance, including optimizing the production process of selenium and producing specific selenium metabolites (Herrero and Wellinger, 2015). There are similarities in selenium metabolism between prokaryotes and eukaryotes. All of them have reported a large number of selenoproteins predicted by bioinformatics, most of which may serve redox functions and play an important role in detoxification, although their functions are unclear. Taking bacteria as an example, there are different metabolic pathways of selenium (Figure 3). Wang et al. have proven the reduction mechanism of selenate and selenite, as well as the mechanism of BioSeNPs and protein synthesis involved in these processes (Wang et al., 2022). The first process is to transport selenate anions to cells through the penetration of sulfate or to cells with the help of oxygen anion transporters (Zolotarev et al., 2008; Aguilar-Barajas et al., 2011). To date, the mechanisms of selenite and selenate reduction have not been fully elucidated. The redox reaction in the second stage mainly includes two reduction mechanisms. One is that the anaerobic microbes living in anaerobic sediments catalyze the dissimilation (respiration) reduction of selenate and selenite to Se^0^. The other is the oxidation of organic substrate or H_2_ coupled with the dissimilatory reduction of selenium oxyanions. Butler et al. showed how selenate is used as a respiratory substrate in detail, and selenium is treated by stabilizing and secreting BioSeNPs (Butler et al., 2012). The final product of these reactions is the red amorphous or monoclinic allotropic modification accumulated in the culture media when selenate or selenite is reduced to Se^0^ under the action of microbes. In other words, the redox reaction begins with reducing selenate to selenite by serine, and then selenite is sent to the cytoplasm *via* an unknown transporter across the cell membrane. Once selenite remains in the cytoplasm, it provides material for the synthesis of BioSeNPs, and they are finally synthesized in the nucleus. The BioSeNPs synthesized by microbes may be covered by surface-related macromolecules, such as proteins, polysaccharides and lipids (Jain et al., 2014; Estevam et al., 2016, 2016; Lampis et al., 2016). The mechanism of selenium immobilization in yeast can be summarized as follows (Figure 4): 1) Se(VI) is transformed into Se(IV) by ATP sulfidase, NADPH, and 3′-phosphoadenylyl selenate reductase; 2) Se(IV) is then converted to Se-glutathione and oxidized glutathione, followed by converting oxidized glutathione to a reducing form *via* glutathione reductase; 3) Se-glutathione is turned into glutathione selenol; and 4) glutathione selenol is transformed into Se (0) and reduces glutathione by superoxide dismutase (Wu et al., 2021).

## 5 Functions of selenium supplementation

**FIGURE 5:**
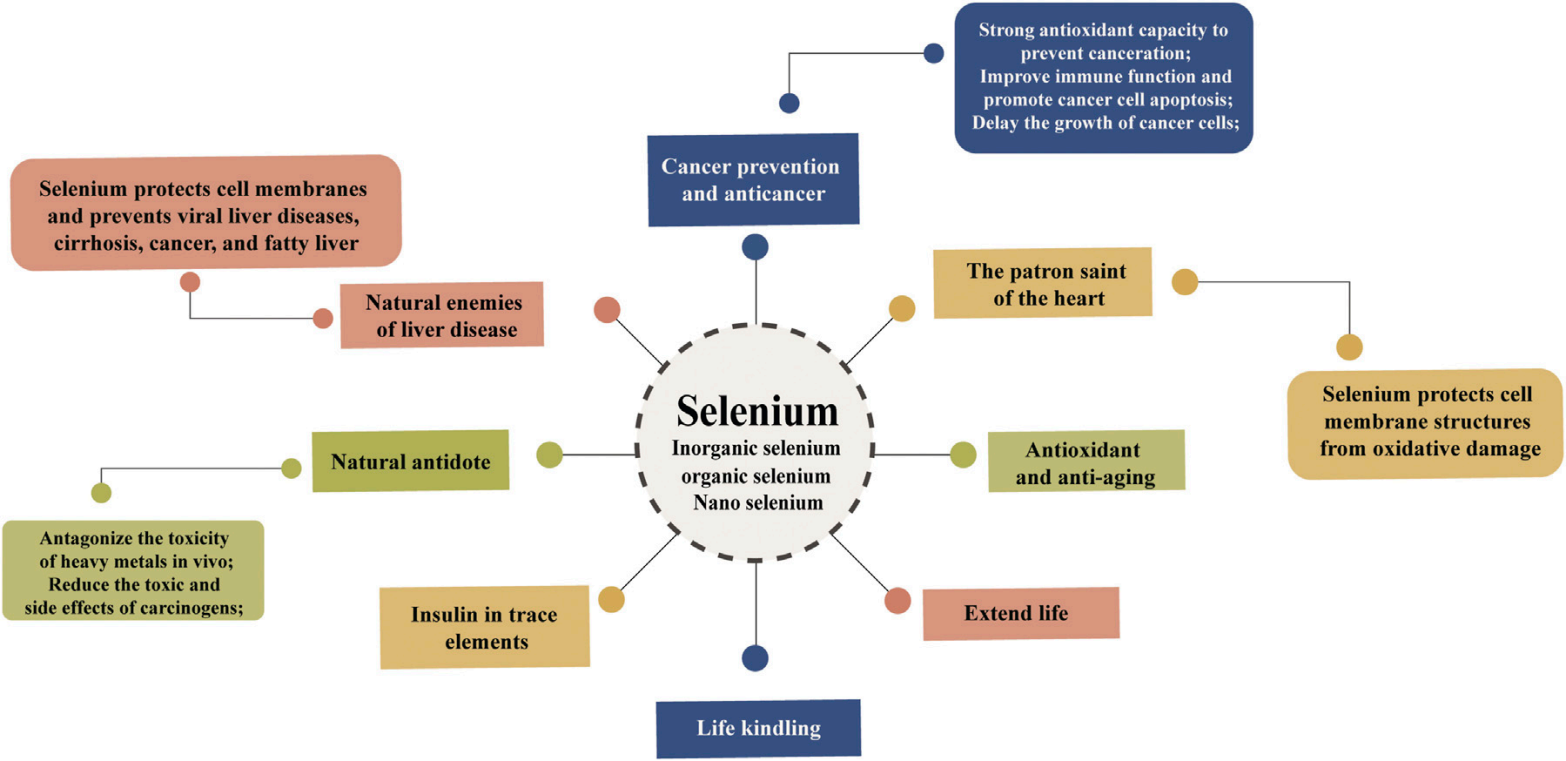
The main functions of selenium in organisms. Selenium plays an important role in the growth of organisms. It has strong antioxidant capacity and is used as a cancer prevention and anticancer agent as well as an antiaging agent. Selenium, which protects cell membrane structures, has been called the enemy of liver disease and the patron saint of the heart. At the same time, selenium can antagonize the toxicity of heavy metals *in vivo* and extend life.

Selenium cannot be synthesized and stored naturally in humans and animals, so external supplementation is necessary to achieve selenium homeostasis, which means that the body’s internal environment and immune system are in balance. In view of the disadvantages of direct dietary inorganic selenium, a series of selenium supplementation strategies have emerged. Dietary selenium is usually supplemented in livestock and poultry nutrition in inorganic (SeO_3_^2-^ and SeO_4_^2-^) or organic forms (SeMet, SeCys, selenopolysaccharide, *etc.*). For quite some time, the toxicity of selenium to animals has been considered, which is the main reason for the long-term alkaline state and physiological disturbance of metabolism of animals. As a component of enzymes (GSH-Px, iodothyronine deiodinase and thioredoxin reductase) and proteins, selenium has a variety of health effects on living individuals. The different forms of selenium ingested biologically also have multiple uses, and selenium supplements can be inorganic selenium, such as Na_2_SeO_4_ or Na_2_SeO_3_, or in the organic selenium form enriched with Se-enriched yeast, SeMet (Gawor et al., 2020; Radomska et al., 2021). BioSeNPs as an emerging selenium supplement. It was verified that BioSeNPs could not only prevent DNA damage but also reduce the mortality of bone marrow cells in mice. In aquatic animal and fish cultures, BioSeNP addition can improve the growth and antioxidant defense systems of fish (Ghaniem et al., 2022). Some characteristics of selenium are attributed to the health advantage of animals and other mammals (Figure 5), such as GSH-Px and thioredoxin reductase, inhibiting viral expression, retarding the aging process, managing oxidative stress, participating in sperm reproduction, developing immunity against causative agents, delaying AIDS symptoms, and preventing some cancers or heart diseases (Pan et al., 2011; Ying and Zhang, 2019; Kieliszek et al., 2022).

**FIGURE 6:**
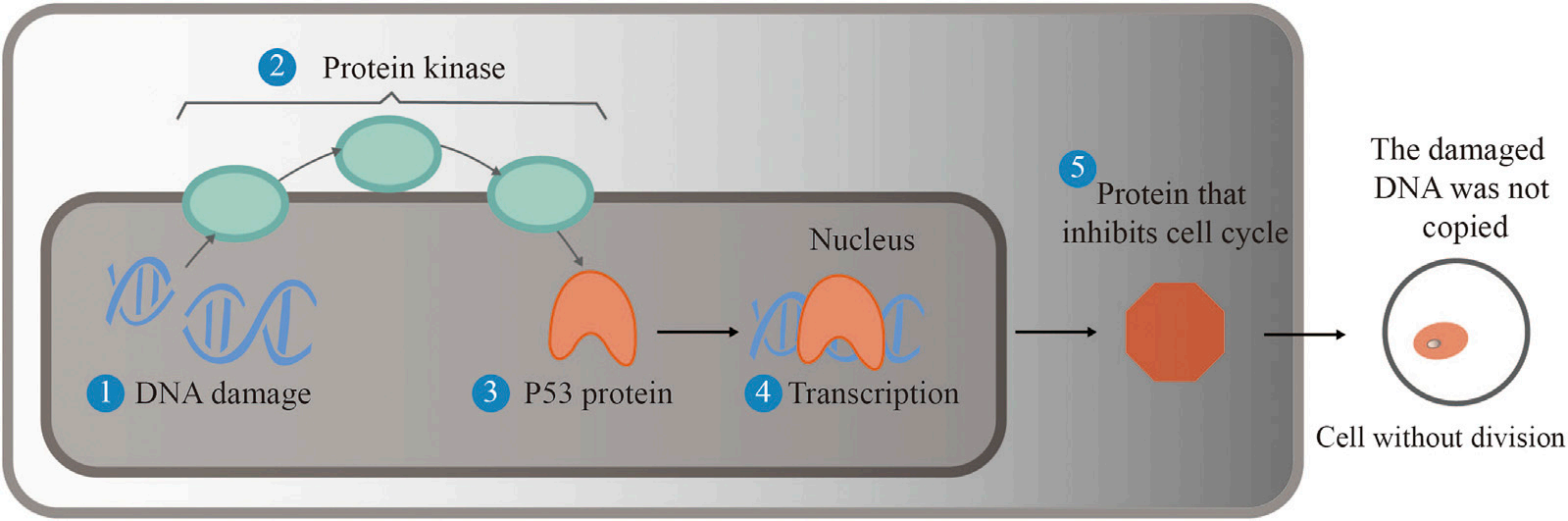
Selenium activates the p53 gene and inhibits cell carcinogenesis. When DNA is damaged, protein kinase activates the p53 gene to translate into a protein that repairs the damaged DNA, thereby halting the cell cycle. If the DNA is seriously damaged, apoptosis is activated to ensure that faulty DNA does not replicate, preventing the cell from becoming cancerous.

As an indispensable cofactor of selenoproteins, selenium plays an important role in improving human reproductive ability and enhancing human immunity and antioxidant function, cardiovascular diseases, and cancer. When selenium is deficient, the body’s immune function is impaired, and it becomes very vulnerable to viral infections. Selenium content in the body affects cellular and humoral immunity (Yang et al., 2021). Experiments in rats supplemented with selenium chitosan have verified that selenium supplementation can improve the immune function of rats and has the ability to block the growth of gastric cancer (Jiang et al., 2021). Therefore, selenium and its signal transduction process lead to differential expression of related genes. Selenium acts as a regulator to maintain homeostasis in both selenium deficiency and supplementation (Xia, 2020; Jiang et al., 2021). Selenium plays an important role in cancer prevention and anticancer function, and this has been widely investigated (Jia et al., 2020). At present, a series of animal experiments have verified the above conclusions. The inhibition mechanism of the p53 gene activated by selenium is shown in Figure 6. The reason why the p53 gene is called the “genome guardian angel” is that when DNA is damaged, p53 is assembled into corresponding proteins to promote the repair of DNA and stop the cell cycle. If the DNA is seriously damaged, the mechanism of apoptosis will be activated, which can ensure that the wrong DNA will not replicate want only; that is, it can inhibit cell carcinogenesis.

Selenite, methylene selenate (DMSe), SeMet and other forms of selenium can activate the p53 gene. Among them, the p53 protein product promoted by SeMet will only promote DNA repair, not stop the cell cycle and cause cell apoptosis to reduce the possibility of cell carcinogenesis (Smith et al., 2004; Andrew et al., 2016). It is obvious that the mechanism of cervical cancer cell death corresponds to the p53 metabolic pathway by selenite. To reduce the risk of colon and rectal cancer, a certain amount of selenium-enriched *S. cerevisiae* was added to animal feed, and this conclusion was verified by experiments in rats (Abedi et al., 2018). Thus, selenium-enriched yeast has potential as an anticancer therapeutic agent (Jiang et al., 2021).

SeNPs can also induce cancer cells to produce reactive oxygen species to activate the degradation and apoptosis of their cancer cell proteins to play a certain anticancer role (Zhao et al., 2020). In addition, selenoprotein has antioxidant and anti-inflammatory effects, which is a simulated effect of insulin (Steinbrenner, 2013). Selenium also has certain benefits to the male and female reproductive systems (Iqbal et al., 2014).

## 6 Detection and characterization of selenium

It is understood that inorganic selenium generally refers to SeO_3_^2-^, SeO_4_^2-^, Se^0^, organic selenium mainly selenocysteine, selenomethionine, selenoprotein, selenocystine, selenium polysaccharide, selenium yeast, SeNPs, and so on. Different forms of selenium have different toxicities to organisms. High selenium concentrations can change and distort protein structure, destroy enzyme structure, and greatly affect organism metabolism. Therefore, quantitative analysis of selenium concentration is of great significance. Many approaches have been carried out on the detection and morphology of selenium. Currently, the quantitative analysis of selenium mainly includes the following methods: UV-vis, atomic fluorescence spectrometry (AFS), fluorescence spectrometry, chromatography, flameless atomic absorption spectrometry, electrochemical methods, and inductively coupled plasma mass spectrometry (ICP-MS) and mass spectrometry (MS) (Pettine et al., 2015; Sharma et al., 2017; Jyothi et al., 2020). There are many contributions to further promote the detection and speciation of selenium in this field.

Due to the low content and complex mechanism of Se in biological tissues, it is necessary to use selective and sensitive analytical techniques for its detection, and ICP-MS is the most effective element-specific method for the determination of total Se in biological samples. The combination of BioSeNPs with organic components (proteins, polysaccharides and lipids) is usually performed by high-performance liquid chromatography (HPLC) combined with ICP-MS for the analysis of selenium morphology (Gawor et al., 2020). Given the size, shape, surface area, surface charge and other characteristics of selenium, a series of traditional modern analytical and biochemical techniques are involved. They are usually characterized by UV-vis, Fourier transform infrared (FT-IR) spectroscopy, scanning electron microscopy (SEM), transmission electron microscopy (TEM), high resolution transmission electron microscopy (HRTEM), energy dispersive X-ray (EDX) analysis, dynamic light scattering (DLS), and X-ray diffraction (XRD). In addition, the presence of SeNPs in cell biomass samples can be determined by X-ray fluorescence (XFA) and electron energy loss spectroscopy (EELS) (Kazemi et al., 2019; Korde et al., 2020).

There are many methods to describe the structure of selenium polysaccharides, including high-performance gel permeation chromatography (HPGPC), gas chromatography (GC), HPLC, methylation, nuclear magnetic resonance (NMR), SEM and AFS (Jamima et al., 2021; Luo et al., 2021). These methods have been performed to determine the molecular weight, glycosidic bond and molecular morphology. There are few studies on the specific binding sites of selenium, and most of them only use FT-IR to confirm the presence of selenium-containing groups. Analysis of Se in Se polysaccharides showed that Se exists in an oxidized state and is organically bound to the polysaccharide structure (Zhou et al., 2020; Zhao et al., 2021).

## Conclusion

Organic selenium combined with biological components (proteins, lipids and polysaccharides), especially selenoproteins, are important nutrients that have been extensively studied and have shown great potential for the development of multiple applications. SeNPs synthesized by biological approaches avoid a series of disadvantages, such as instability, low bioavailability, and high toxicity, compared with physical and chemical methods. BioSeNPs can be extracted by biological methods and characterized by UV-vis, FT-IR, SEM, TEM, HRTEM, EDX, DLS, and XRD analysis, and these methods are explored to characterize the size, morphology and potential applications. To promote practical applications, more research should be conducted to clarify the synthetic mechanism of protein- or polysaccharide-modified BioSeNPs and to develop yeast resources with higher selenium accumulation and conversion.
